# Pre-Treatment Methods for Regeneration of Spent Activated Carbon

**DOI:** 10.3390/molecules25194561

**Published:** 2020-10-06

**Authors:** Sang Youp Hwang, Gi Bbum Lee, Ji Hyun Kim, Bum Ui Hong, Jung Eun Park

**Affiliations:** Bio Resource Center, Institute for Advanced Engineering, Yongin-si 17180, Korea; syhwang80@gmail.com (S.Y.H.); mnbbv21c@gmail.com (G.B.L.); jhkim2017@iae.re.kr (J.H.K.); buhong@iae.re.kr (B.U.H.)

**Keywords:** spent activated carbon, regeneration, chemical activation, acid solution, pre-treatment, high surface area

## Abstract

Spent activated carbon (SAC) usually exhibits a low specific surface area due to its high ash contents. In this study, pre-treatments, such as heat and acid treatments, were optimized to improve this feature. The heat pre-treatment did not reduce the ash content, nor did it increase the surface area. Because metallic ions adsorbed in SACs turn into ash upon the heat treatment. In the acid pre-treatment, the volatiles and fixed carbon were increased with decreasing ash contents. In this study, it was found that the surface area increase was correlated with the ratio between fixed carbon and ash. Among the pre-treatment methods, the combined heat and acid pre-treatment method highly increased the ratio, and therefore led to the surface area increase. Additionally, the acid pre-treatment was carried out using different types of acid (organic and inorganic acids) solutions to further improve the surface areas. The organic acid treatment caused a significant structural collapse compared to the inorganic acid treatment, decreasing the surface area. In particular, H_3_PO_4_ effectively removed ashes adsorbed on the activated carbon surface and regenerated the exhausted activated carbon. Both the heat and acid pre-treatments before chemical activation resulted in the positive effects such as strong desorption of pollutants and ashes within the internal structure of the activated carbon. Therefore, the regeneration introduced in this study is methodically the best method to regenerate SAC and maintain a stable structure.

## 1. Introduction

Drinking water guidelines of the World Health Organization (WHO), which are followed by South Korea [1], define permissible levels of contaminants in water. Adsorption is one of the most effective methods to remove contaminants from wastewater due to its simple operation, low cost, and high adsorption efficiency. Accordingly, activated carbon (AC) is commonly used as an adsorbent in water and wastewater treatment facilities because it can adsorb a wide variety of organic and inorganic contaminants due to its porous nature, surface area, and abundant surface functional groups [2,3,4]. Therefore, consumption of ACs for industrial use has become an indicator of development and environmental management efficiency. ACs are generally prepared using natural resources, such as wood, coal, nutshells, and petroleum residues [5,6,7]. However, the price of precursors, especially woods, has recently increased due to restricted logging, which also commonly causes environmental impacts. Thus, the treatment of spent adsorbents is one of the most viable options to effectively solve the critical problems in view of environmental and economic considerations than disposal in landfill and reutilization [8,9,10]. The waste resources were easily accessed, but complex production processes should be required. In contrast, if the SACs are used as resources for the AC production, additional carbon precursor is not required and also the only regeneration process is required. In this study, the regeneration of SAC has been focused owing to the environmental protection and low production cost. Supplementary Materials Table S1 shows the cost comparison of regenerated and fresh ACs. The cost of regenerated ACs was 35–55% lower than the fresh ones [11]. Additionally, the various regeneration methods were introduced, and also summarized their both advantages and disadvantages in Supplementary Materials Table S2. Among the methods, the landfilling was inexpensive and simple method. However, it caused the serious environmental problems, likely soil contamination. Considering the environmental protection and production cost, the regeneration of SACs was suggested. The suitable method also should be selected for the efficient regenerations of SACs.

The replacement cycle of AC is commonly between three to four years, and the used AC is known as SAC. Compared to AC, the surface area of SAC is approximately 30–40% lower, and the ash content is approximately 10% higher [12]. Most water treatment plants prefer to replace new AC instead of reusing SAC, despite the high price of the former. The main reasons for this choice are: (1) adsorbed materials still remain in regenerated AC after heat activation, (2) the regenerated AC presents inferior physical properties, and (3) regeneration effect rapidly decreased.

The surface area of regenerated SACs by thermal process can be recovered about 60% and the carbon content decreased during regeneration process, but that is only commercially viable technique [13]. Also, the ash content was increasing from 1.6 to 3.0 times, thus decreasing the SAC structural stability. A high ash content significantly decreases the activity and regeneration efficiency of SAC. Therefore, regeneration methods should be further developed to improve the physical properties of SAC [14].

To improve the regeneration efficiency, various pre-treatment methods currently have been developed. Among the several pre-treatments, an acid treatment is commonly suggested to remove organic materials. However, the acid pre-treatment causes the oxygen functional group of the AC to collapse or etch over the oxygen functional group, and it can affect the final surface area [15]. Moreover, the acidic groups form acidic oxygen surface complexes, which can affect their use as adsorbents or as catalysts [16,17]. Thus, regeneration of physical properties by such treatments (pre-treatment, leaching, or washing) should be optimization.

The aim of this research is to evaluate the regenerated SAC by the different pre-treatment methods. The present research evaluates the influence of experimental parameters such as pre-treatment pathway and acidity groups on the characteristics of regenerated SAC. In particular, the surface area of SACs after different pre-treatments was compared, and the correlation between surface area and physical properties was investigated.

## 2. Materials and Methods

The SACs were obtained from a wastewater treatment plant at Seoul in South Korea. Before use, the SACs were dried at 100 °C for 24 h. To confirm the effects of the acid pre-treatment, organic or inorganic acid solutions were used. For the organic acid pre-treatment, citric acid (C_6_H_8_O_7_, Sigma-Aldrich, MA, USA, >99%), oxalic acid hydrate (C_2_H_2_O_4_, Sigma-Aldrich, MA, USA, 99%), and DL-tartaric acid (C_4_H_6_O_6_ H_2_O, Duksan, Ansan, South Korea, >99.5%) were selected. For the inorganic acids, hydrochloric acid (HCl, Daejung, Incheon, South Korea, 35–37%), hydrogen peroxide (H_2_O_2_, Duksan, Ansan, South Korea, 28%) [18], and phosphoric acid (H_3_PO_4_, Sigma-Aldrich, MA, USA, ACS reagent >85% in H_2_O) were selected.

The SACs were prepared by four types of pathways, named Case 1, 2, 3, and 4, and heat treatment, acid treatment, and chemical activation were investigated.

**Figure 1:**
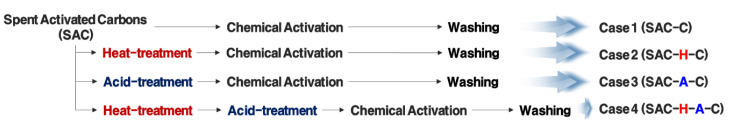
The various methods for regenerating activated carbon.

For Case 1, the treatment method was a one-step chemical activation. The solid phase of the SAC was mixed with a solid phase of potassium hydroxide (KOH, Samchun Chemical, Seoul, South Korea), at a SAC/KOH weight ratio of 0.5 [19,20,21]. The samples were gradually heated at 5 °C/min until 750 °C, at which the temperature was maintained for 1 h. Subsequently, the samples were heated to 850 °C at a rate of 5 °C/min, and then held for 3 h. After chemical activation, the samples were washed with water three times and then neutralized. This sample was named SAC-C. For Case 2, approximately 10 g of SAC was slowly heated in a quartz reactor at a heating rate of 5 °C/min from room temperature to 500 °C under a nitrogen (N_2_) atmosphere (flow rate of 100 mL/min) [22]. The samples were held at this temperature for 10 h before slowly cooling down under a N_2_ atmosphere. After the heat treatment, the SAC was activated with KOH in the same manner as in Case 1. This sample was named SAC-H-C. For Case 3, the SACs were treated by organic and inorganic acids before chemical activation. For that, 10 g of SAC was washed 0.5 M acid solution for 0.5 h, filtered and rinsed with deionized water (DI) until neutral pH was achieved, and then dried at 105 °C in an oven for approximately 24 h. After the acid pre-treatment, the chemical activation was the same as that for Case 1 (SAC-A-C). In Case 4, both previous pre-treatments (heat and acid treatments) were used, after which chemical activation and washing methods were performed (denoted as SAC-H-A-C). Figure 1 describes the SAC regeneration pathway in Cases 1–4.

For the proximate analysis, dried SAC samples were placed in a furnace (Daeheung Sci., DF-4S, Incheon, South Korea) and heated at 950 °C for 7 min, and then 750 °C for 10 h. The ash, volatile s, and fixed carbon contents within the SACs were measured as weight percentages.

An elemental analysis (EA) was conducted by following procedures described elsewhere using an elemental analyzer (Flash EA 1112, Thermo Scientific, Milan, Italy). The elemental contents of C, H, O, N, and S were determined [19,20].

The surface area of SACs was analyzed using the Brunauer-Emmett-Teller (BET) method based on the N_2_ adsorption at −196 °C using an adsorption analyzer (ASAP 2010, Micrometrics, Norcross, GA, USA). The pore size of the SAC was calculated according to the Barrett Joyner and Halenda (BJH) method. Before carrying out the N_2_ isotherms, the SAC were outgassed at 350 °C to a constant vacuum (P/P_o_ = 2µmHg) for 6 h.

The materials adsorbed on the SACs were analyzed by X-ray fluorescence (XRF-1800, Shimadzu, Tokyo, Japan).

## 3. Results and Discussion

### 3.1. Regeneration of Pre-Treated SAC

As mentioned in the experimental section, different pre-treatments were conducted to identify the most efficient process for SAC regeneration. The surface area and pore size distribution of SAC were 681.59 m^2^/g and 3.39 nm. The chemical activation efficiently enhanced the surface area of the samples, but the ash generation was observed after the activation, as shown in Supplementary Figure S1. The adsorbed metal ions on the ACs were not eliminated during the regeneration process (especially thermal and chemical activation), and then it was remained on the carbon surface and pores. The metal ions adsorbed on the ACs were reduced and converted into the ashes during the thermal process, which also could block the pores [21]. In previous reports, acid washing is widely used after chemical activation to remove the ashes from the SAC pores. In the present study, SAC was chemically regenerated, but post-treatments such as neutralization using acid solution were not conducted. After the activation, the samples were simply washed with DI water to confirm the pre-treatment effects.

Phosphoric acid was used as the strong acid in the pre-treatment process (Figure 1). Table 1 shows the AC properties obtained upon different pre-treatment processes. The SAC-C without pre-treatments exhibited higher specific surface area than those with pre-treatments. Although the surface area increased, the ash content in ACs before and after chemical activation (i.e., SAC and SAC-C, respectively) was comparable, as shown in Supplementary Figure S2. This result indicates that the generated ashes could not be eliminated without the additional acid washing. The potassium (K) only increased in SAC-C due to the KOH activation process. The various pre-treatment processes were carried out in this study, and the reason to choose the process was summarized in Supplementary Table S3 [23,24]. The expected effects and actual results were also compared. The expectation of heating and acid washing was removal of ashes and structure preventing, respectively. In the pre-treated ACs, the surface area increased in the following order: SAC-H-C < SAC-A-C < SAC-H-A-C. Furthermore, SAC-H-C, which was thermally pre-treated, exhibited lower surface areas due to the generated ashes, as previous research [25]. To reduce the ash load, an acid pre-treatment was conducted for the SAC-A-C. The SAC was treated by phosphoric acid (H_3_PO_4_) prior to the chemical activation to eliminate the adsorbed metal ions which caused the ashes. The process effectively reduced the ash production (6.1%), as shown in Table 1, but it did not significantly affect the specific surface area. It might be a possible explanation that higher concentration of acid aqueous will deteriorate the SAC by diminishing heavy metal desorption [15]. The SAC-H-A-C was treated with both heat and acid pre-treatments before the activation process. The amount of generated ash was reduced, and the specific surface area was comparable to that of SAC-C. The pre-treatment using both heat and acid treatments was more efficient than either method alone. The combined treatment of both thermal air and oxalic acid aqueous washing is the most excellent method for recovering the textural properties of SAC than one step [8]. However, the resulting surface area (SAC-H-A-C) was slightly lower than that of SAC-C.

**Figure 2:**
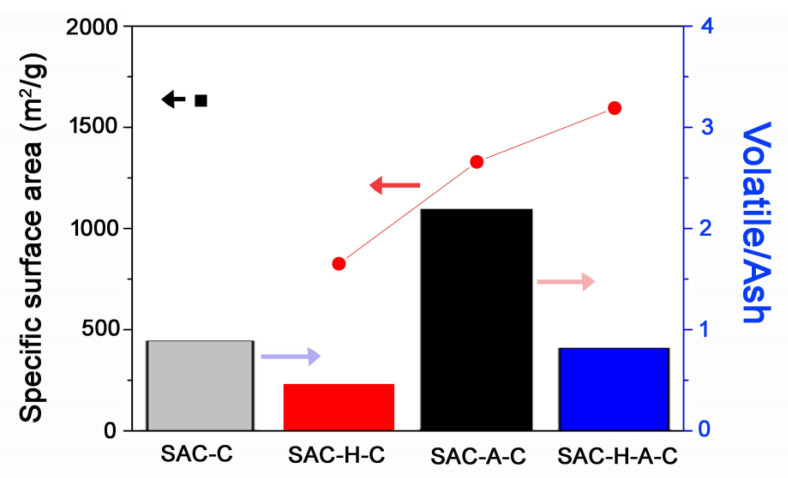
Relationship between surface area and ratio of volatile/ash at different pre-treatment methods. The square (▪) and circle (•) symbols indicated the specific surface areas (left *y*-axis), and the SAC-C(**gray**), SAC-H-C(**red**), SAC-A-C(**black**), and SAC-H-A-C(**blue**) bars represented the ratio of volatile/ash (right *y*-axis).

The AC obtained after the thermal pre-treatment exhibited lower volatile contents. This occurred because the process initially eliminated the oxygen functional groups from the sample surface. The specific functional groups representing the volatile compounds possibly enhanced the specific surface areas [26], but they did not significantly affect the results, as shown in Table 1. In addition, the ash amount usually affects the surface area increase [27]. The generated ashes cover the carbon active sites, which prevents the activation that increases the surface area. To confirm the effect of volatile and ash, their correlation between their ratio and surface areas are shown in Figure 2. The surface area and related ratio of volatile/ash of SAC-C and SAC-H-A-C were similar. The ratio of both samples was relatively low which indicated a lower amount of volatile and higher amount of ash content, and their surface area was comparatively high. In this case, the changes were mainly caused by the amount of volatile. Therefore, it can be concluded that the volatile/ash ratio was not directly correlated with specific surface areas.

### 3.2. Acid Pre-Treatment Effect

**Figure 3:**
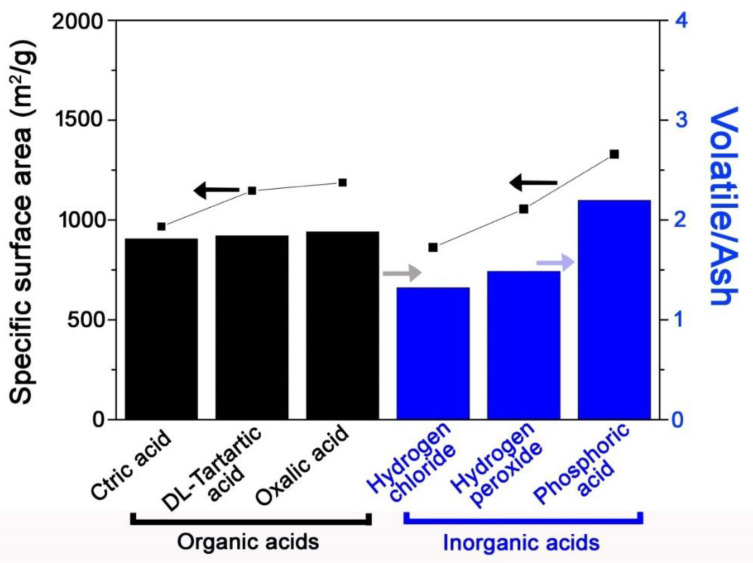
Relationship between surface area and ratio of volatile/ash using the acid pre-treatment methods. The square (▪) symbols indicated the specific surface areas (left *y*-axis), and the both **black** and **blue** bars represented the ratio of volatile/ash (right *y*-axis).

The specific surface area and proximate results of the ACs after the various acid pre-treatments are summarized in Table 2 with Supplementary Figure S3. The surface area of SAC-A-Cs after the acid pre-treatment was higher than that of SAC-H-C (825.40 m^2^/g). Most SAC-A-Cs, except the one treated with H_3_PO_4_, exhibited similar volatile and ash contents, and the H_3_PO_4_ pre-treatment reduced their contents. The ratio of volatile and ash vs. specific surface area are shown in Figure 3. The samples pre-treated with organic acids exhibited similar ratios, and the difference among their surface areas was within 5%. In contrast, the volatile/ash ratio of the samples pre-treated with inorganic acids increased in the following order: HCl < H_2_O_2_ < H_3_PO_4_. Particularly, the H_3_PO_4_ pre-treatment significantly increased the ratio, thus indicating that the ash content was sharply reduced, which led to the increase of specific surface area. An efficient ash removal is essential to increase the SAC surface area. Accordingly, the SAC-A-C with H_3_PO_4_ treatment, which exhibited the highest volatile/ash ratio and lowest ash content, presented the highest surface area increase. However, its surface area was still lower than that of SAC-C.

### 3.3. Combination of Heat and Acid Pre-Treatment

**Figure 4:**
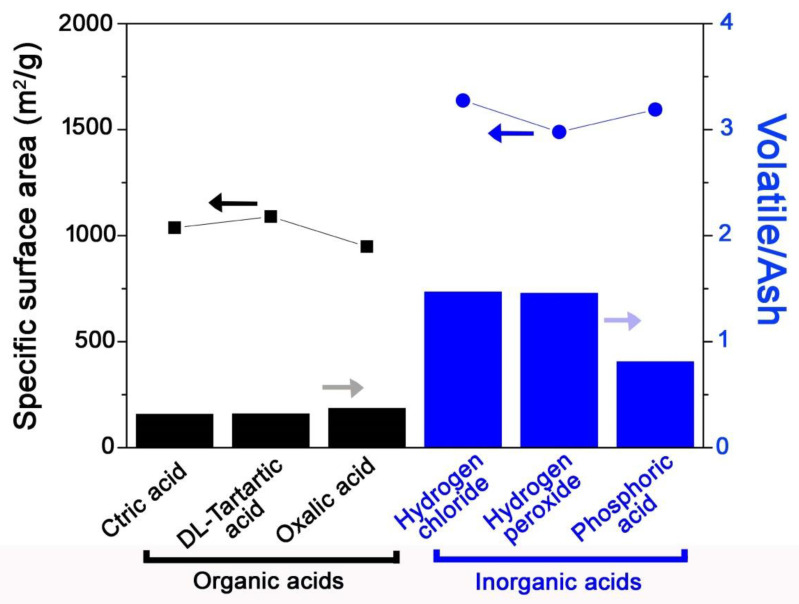
Relationship between surface area and ratio of volatile/ash using the combination pre-treatment (heat and acid) methods. The square (▪) and circle (•) symbols indicated the specific surface areas (left *y*-axis), and the both **black** and **blue** bars represented the ratio of volatile/ash (right *y*-axis).

As shown in Table 1 and Figure 2, the combined heat and acid pre-treatment increased the surface area of the samples. SAC was thermally treated and then sequentially washed by an acid solution. After both pre-treatments, the SAC-H-A was chemically regenerated (activated). During the thermal treatment, the adsorbed metal ions were converted into the ashes, which led to pore blockage. However, the ashes were efficiently removed by the acid solutions, which likely improved the pore characteristics, including surface areas. To optimize the pre-treatment process, various acids were used, and the obtained surface areas were compared. The SAC-H-A-Cs properties are summarized in Table 3 and Supplementary Figure S4. The surface areas of SAC-H-A-C after inorganic acid pre-treatments were approximately 50% higher than those after organic acid pre-treatments. In addition, the organic treatments reduced the contents of volatile but also increased their ash contents. In contrast, the inorganic treatment reduced their ashes, but it did not eliminate the volatiles. Inorganic acid treatments also led to lower oxygen contents compared with the organic treatments. The results shown in Table 3 indicate that the inorganic acid efficiently removed the generated ashes from the heat treatment, thus leading to higher surface areas. Figure 4 also shows the correlation between the ratio of volatile/ash and specific surface area. The ratio for SAC-H-A-Cs after inorganic acid pre-treatment was higher than those for organic treated samples, which also represented the higher surface areas. Particularly, the H_3_PO_4_ pre-treatment reduced both volatiles and ash contents, which is similar to the results described in Section 3.2, thus leading to a ratio decrease, as shown in Figure 4. The amount of ash was more likely to affect the surface areas than the number of volatile adsorbates. The results confirm that the ash contents significantly affect the samples’ surface areas, and H_3_PO_4_ efficiently removed both adsorbed metal ions and generated ashes in the pre-treatment stages, thereby leading to higher surface areas. Therefore, it is possible that the generated ashes were correlated with specific surface areas.

### 3.4. Pre-Treatment Effects

**Figure 5:**
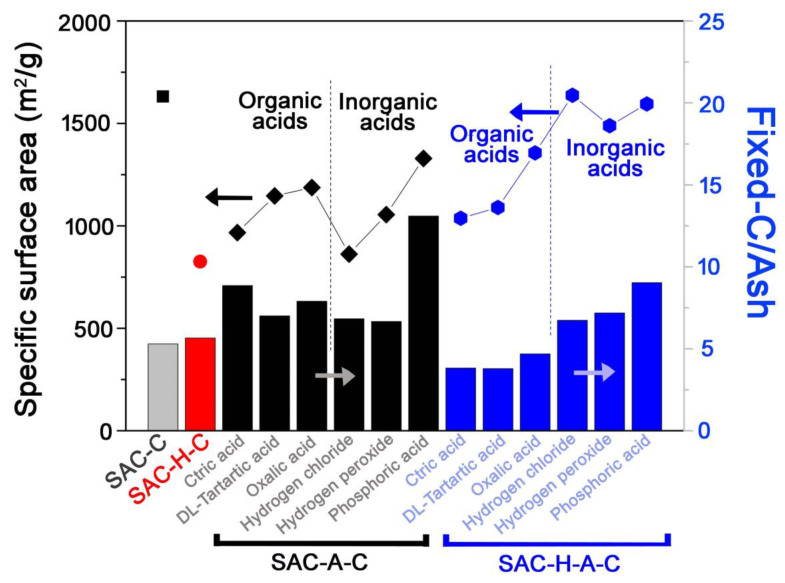
Correlation between surface area and ratio of fixed-C/ash at different pre-treatment methods. The symbols indicated the specific surface areas (left *y*-axis), and the SAC-C(**gray**), SAC-H-C(**red**), SAC-A-C(**black**), and SAC-H-A-C(**blue**) bars represented the ratio of fixed-C/ash (right *y*-axis).

Figure 5 shows the relation between ash content and specific surface area. As mentioned above, the ash content significantly influenced the surface area, so their correlation under different pre-treatments was investigated. For a qualitatively comparison, the fixed-C/ash ratio was used, as shown in Figure 5. Overall, as the ratio increased, the surface area also increased.

Compared to other samples, the fixed-C/ash ratios in SAC-C and SAC-H-C were low, which indicated a large ash generation. In contrast, the ratio increased for samples that underwent acid pre-treatments (SAC-A-C and SAC-H-A-C). The fixed-C/ash ratio of SAC-A-C was higher than that of SAC-H-A-C, but their surface areas were not proportional. The acid pre-treatment in SAC-A-C eliminated the adsorbed metal ions, which could convert into ashes, but it simultaneously collapsed the carbon structure. Therefore, although the acid pre-treatment efficiently removed the ash, it negatively affected the surface area increase. The SAC-H-A-C, which contained both heat and acid pre-treatments, presented a lower fixed-C/ash ratio, but their surface increased. The thermal pre-treatment led to a partial graphitic structure [28], which prevented the formation of the carbon structure during the acid treatment. Moreover, the inorganic pre-treatment efficiently removed the ashes, thus leading to surface area increase. In addition, the advantage of SAC-H-A-C was the generation of high specific surface area, comparing with the previous results (Supplementary Table S4) [29,30,31]. The enhanced surface areas could increase the adsorption capacity, which also raise the product value.

According to previous reports, acid washing after chemical activation (post-treatment) can remove the generated ashes, thus leading to higher surface areas. If this acid post-treatment would be applied to the SAC-H-A-C samples of this study, the remaining ashes after the activation would likely be eliminated, thus further increasing their surface areas. The SAC-C exhibited the highest surface areas and also required the simplest process, which leads to economic benefits. The SAC-H-A-C also exhibited surface area comparable to that of SAC-C, but it required a complex process that demands additional energy for the heat treatment and produces waste acid solutions. Therefore, direct regeneration of SAC using only chemical activation would be suggested based on the results of this study.

## 4. Conclusions

SAC was regenerated using various treatment methods, namely heat, acid, and activation treatments. The chemical activation was already known as a simple method to regenerate SACs. In this study, different treatments were combined to further improve the properties (i.e., surface area) of SAC. Usually, the main limitation for the regeneration of SAC is the elimination of ashes from their pores. Therefore, acid washing was used as a pre-treatment process to remove the ashes prior to chemical activation. Four regeneration pathways (i.e., SAC-C, SAC-H-C, SAC-A-C, and SAC-H-A-C) were investigated, and their properties were compared. The surface areas increased in the following order: SAC-C > SAC-H-A-C > SAC-A-C > SAC-H-C. Among the pre-treated ACs, SAC-H-A-C exhibited the highest surface areas. The use of only heat or acid pre-treatment (i.e., SAC-H-C and SAC-A-C) resulted in a negative effect on the samples’ surfaces. In contrast, the SAC-H-A-C, to which both heat and acid pre-treatments were applied, led to an efficient pore development and increase of fixed carbon ratio, thus resulting in higher surface areas. Particularly, the inorganic acid was more efficient for ash removal than the organic acid. The ash removal increased the fixed-C/ash ratio, and there was a linear correlation between surface areas and fixed-C/ash.

The SAC-H-A-C samples exhibited higher surface areas, which were comparable to that of SAC-C. The pre-treatment of SAC-H-A-C required additional energy and acid solution, while the direct regeneration of SAC-C only required a chemical activation process. For a simpler process with economic benefits, the regeneration method used for SAC-C would be suggested.

## Figures and Tables

**Table 1:** Pre-treatment effect of regenerated SACs.

		SAC	SAC-C	SAC-H-C	SAC-A-C	SAC-H-A-C
BET surface area (m^2^/g)		681.59	1631.70	825.40	1329.00	1594.76
Proximate analysis (%)	Volatile	13.85	12.40	6.46	13.48	7.48
	Fixed-C	71.28	73.70	79.49	80.38	83.30
	Ash	14.86	13.90	14.04	6.14	9.22

**Table 2:** Specific surface area and proximate results of regenerated ACs with acid pre-treatment effect using organic and inorganic acids (SAC-A-C).

		Organic Acid			Inorganic Acid		
		C_6_H_8_O_7_	C_4_H_6_O_6_	C_2_H_2_O_4_	HCl	H_2_O_2_	H_3_PO_4_
Specific surface area (m^2^/g)		967.19	1146.24	1187.27	836.87	980.67	1329.37
Proximate analysis (%)	Volatile	15.52	18.69	17.44	14.43	16.23	13.48
	Fixed-C	75.91	71.16	73.29	74.65	72.85	80.38
	Ash	8.57	10.15	9.27	10.92	10.92	6.14

**Table 3:** Specific surface area and proximate results of regenerated ACs with the combined the heat and acid pre-treatment effect using organic and inorganic acids (SAC-H-A-C).

		Organic Acid			Inorganic Acid		
		C_6_H_8_O_7_	C_4_H_6_O_6_	C_2_H_2_O_4_	HCl	H_2_O_2_	H_3_PO_4_
Specific surface area (m^2^/g)		1037.10	1089.90	948.10	1637.42	1489.06	1594.76
Proximate analysis (%)	Volatile	6.15	6.26	6.14	15.97	15.11	7.48
	Fixed-C	74.4	74.14	77.34	73.25	74.55	83.30
	Ash	19.46	19.59	16.51	10.78	10.37	9.22
Ultimate analysis (%)	Carbon	79.24	79.99	77.53	83.21	84.75	87.83
	Hydrogen	1.06	1.37	1.18	1.02	0.77	1.06
	Oxygen	17.19	16.18	18.86	12.17	12.48	9.66
	Nitrogen	0.55	0.61	0.27	0.92	0.54	0.29
	Sulfur	0.05	0.06	0.06	1.33	0.07	0.08

## Supplementary Materials

The following are available online, Table S1: Cost comparison of reactivated and virgin carbon, Table S2: Advantage, disadvantage, and operating cost of regenerated SACs, Table S3: Hypothesis and experimental results of regenerated SACs, Table S4: Summary of specific surface area of regenerated ACs, Figure S1: X-ray fluorescence (XRF) analysis of the chemical composition of SAC(green), SAC-C(gray), and SAC-H-A-C(blue) bars, Figure S2: Nitrogen adsorption and desorption isotherms of (a) SAC, (b) SAC-C, (c) SAC-H-C, Figure S3: Nitrogen adsorption and desorption isotherms of SAC-A-Cs; (a) C_6_H_8_O_7_, (b) C_4_H_6_O_6_, (c) C_2_H_2_O_4_, (d) HCl, (e)H_2_O_2_, (f) H_3_PO_4_, Figure S4: Nitrogen adsorption and desorption isotherms of SAC-H-A-Cs; (a) organic acids; C_6_H_8_O_7_, C_4_H_6_O_6_, C_2_H_2_O_4_, (b) HCl, H_2_O_2_, H_3_PO_4_.
