# Supplementary material for: Pre-Treatment Methods for Regeneration of Spent Activated Carbon
Source: Molecules. 2020 Oct 6;25(19):4561. doi: 10.3390/molecules25194561 (PMC7584032; doi:10.3390/molecules25194561)
Supplement: Supplementary file 1 [file molecules-25-04561-s001.pdf]

[Supporting Information]

Pre-treatment methods for regeneration of spent activated carbon

Sang Youp Hwang[a,‖], Gi Bbum Lee[a,‖], Ji Hyun Kim[a], Bum Ui Hong[a], Jung Eun Park[a,*]

[a]*Bio Resource Center, Institute for Advanced Engineering, Yongin 17180, Korea*

‖ S.Y.H and ‖ G.B.L. contributed equally to this manuscript

* Corresponding author. Tel: +82-31-330-7274

E-mail address: jepark0123@gmail.com

Table S1. Cost comparison of reactivated and virgin carbon [11].

	Regional Reactivation	Virgin Carbon
**Utility**	**$/lb**	**$/lb**
**Connecticut**	0.96	1.46
**Danvers, MA**	0.55	0.92
**Lowell, MA**	0.65	1.46

[11] Hamura,D.; Sagayaga, A.; Babcock, R. Literature review of regeneration methods and local disposal alternatives, 2018, Water Resources Research Center, University of Hawaii. https://scholarspace.manoa.hawaii.edu/bitstream/10125/22235/WRRC-98-12.pdf

Table S2. Advantage, disadvantage, and operating cost of regenerated SACs [11].

Regeneration type	Advantage	Disadvantage
**Landfill**	▪ Inexpensive method<br>(No capital / maintenance cost)	▪ Environmental pollution
**Thermal regeneration**	▪ Simple regeneration process	▪ High energy requirement<br>▪ Low yield / Pore structure change<br>▪ Air pollution
**Solvent regeneration**	▪ In-situ recycling (possible)<br>▪ High regeneration efficiency	▪ Danger from hazardous chemicals<br>▪ High cost of reagents
**Wet oxidation**	▪ Low energy consumption<br>(temperature / pressure)	▪ Low regeneration efficiency

[11] Hamura,D.; Sagayaga, A.; Babcock, R. Literature review of regeneration methods and local disposal alternatives, 2018, Water Resources Research Center, University of Hawaii.
https://scholarspace.manoa.hawaii.edu/bitstream/10125/22235/WRRC-98-12.pdf

Table S3. Hypothesis and experimental results of regenerated SACs.

	Hypothesis (expected effect)	Specific surface area (m^2/g)	Experimental result
**SAC-H-C**	▪ Eliminate adsorbates [23]	825	▪ Low surface area<br>▪ Ash generation/pore blockage
**SAC-A-C**	▪ Efficient ash removal [24]	1329	▪ Ash removal<br>▪ Structure collapse
**SAC-H-A-C**	▪ Structure prevent<br>▪ Efficient ash removal	1595	▪ Low ash contents<br>▪ High surface area

[23] Miguel, G.S.; Lambert, S.D.; Graham, N.J.D. Thermal Regeneration of Granular Activated Carbons Using Inert Atmospheric Conditions. *Environmental Technology* **2002**, 23, 1337-1346. DOI: 10.1080/09593332508618449

[24] Van der Bruggen B.; Vogels, G.; Van Herck, P.; Vandecasteele, C. Simulation of acid washing of municipal solid waste incineration fly ashes in order to remove heavy metals. *Journal of Hazardous Materials* **1998**, 57, 127-144. DOI: 10.1016/S0304-3894(97)00078-2

Table S4. Summary of specific surface area of regenerated ACs.

	Specific surface area (m^2/g)		Reference
**SAC-H-A-C**	1595	Thermal/Chemical activation	This study
**Regenerated AC**	1033	Microwave heating	[29]
**RWPAC (regeneration of waste powder AC)**	1161	Pyrolysis	[30]
**SA (sintering-activation)**	1001	Thermal regeneration	[31]

[29] Xia, H.; Wu, J.; Xia, Y.; Zhang, L.; Peng, J.; Wang, S.; Zheng, Z.; Zhang, S. Microwave assisted regeneration of spent activated carbon from petrochemical plant using response surface methodology. *Journal of Porous Materials* **2015**, *22*, 137–146. DOI: 10.1007/s10934-014-9880-x.

[30] Li, Y.; Jin, H.; Liu, W.; Su, H.; Lu, Y.; Li, J. Study on regeneration of waste powder activated carbon through pyrolysis and its adsorption capacity of phosphorus. *Scientific Reports* **2018**, *8*, 778. DOI: 10.1038/s41598-017-19131-x.

[31] Cho, J.-H.; Kim, Y.-S.; Jeon, S.-B.; Seo, J.-B.; Jung, J.-H.; Oh, K.-J. Improvement of thermal regeneration of spent granular activated carbon using air agent: Application of sintering and deoxygenation. *Korean Journal of Chemical Engineering* **2014**, *31*, 1641-1650. DOI: 10.1007/s11814-014-0125-0.

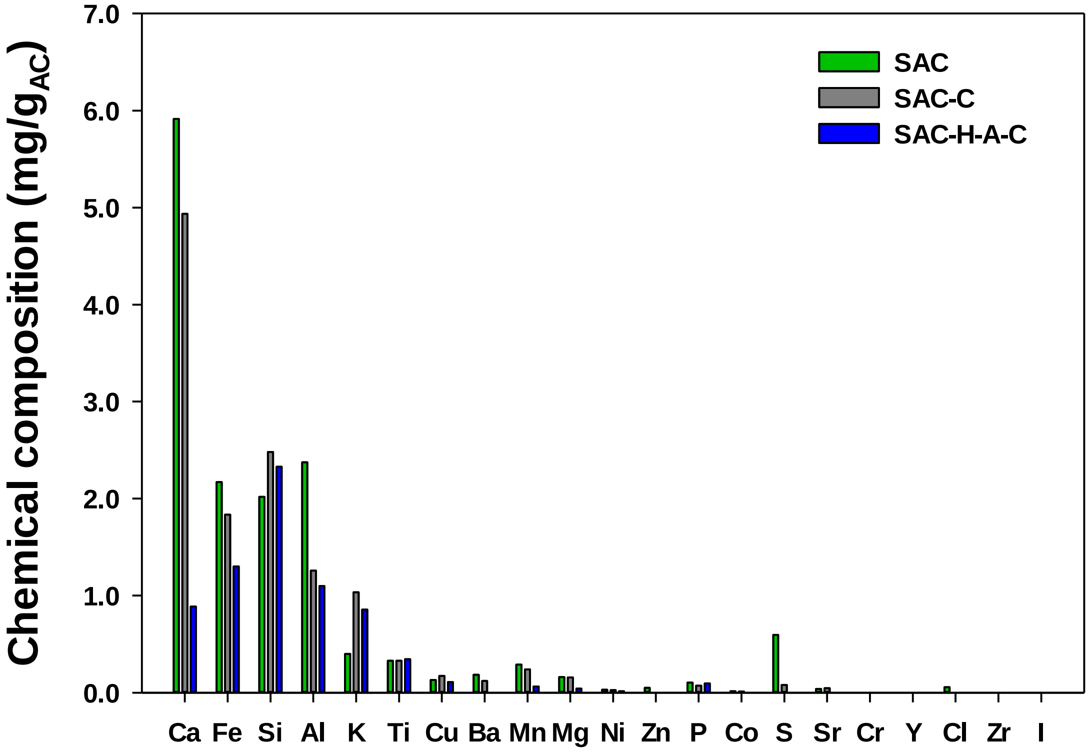

Figure S1. X-ray fluorescence (XRF) analysis of the chemical composition of SAC(green), SAC-C(gray), and SAC-H-A-C(blue) bars.

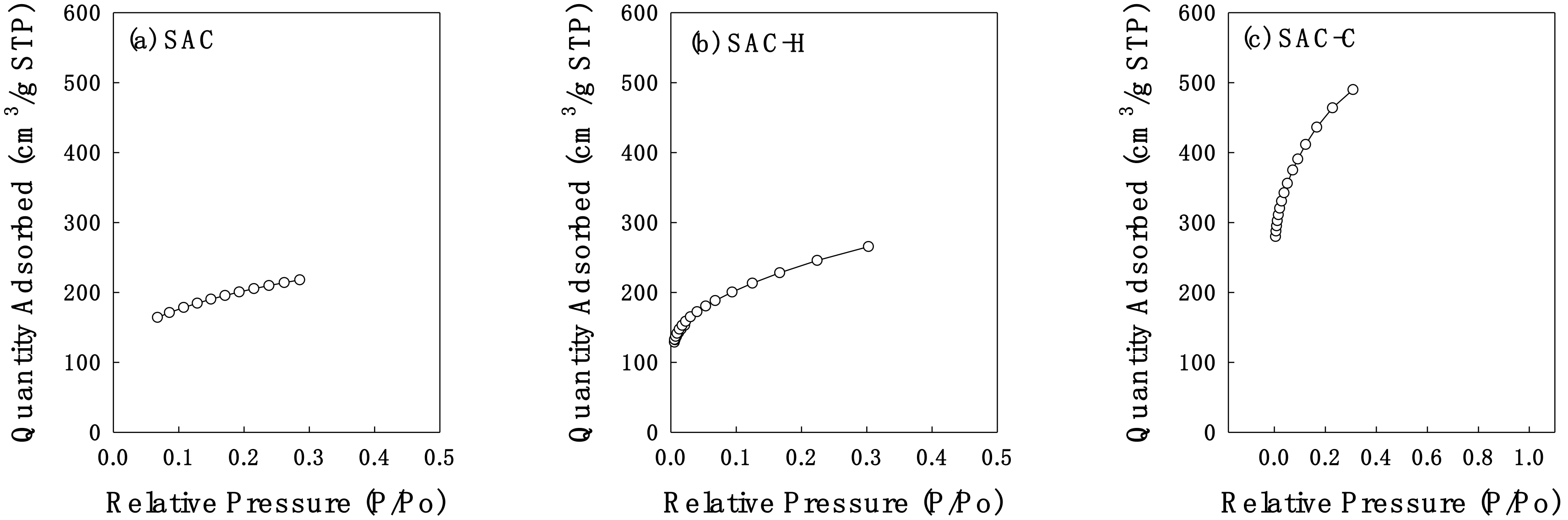

Figure S2. Nitrogen adsorption and desorption isotherms of (a) SAC, (b) SAC-C, (c) SAC-H-C.

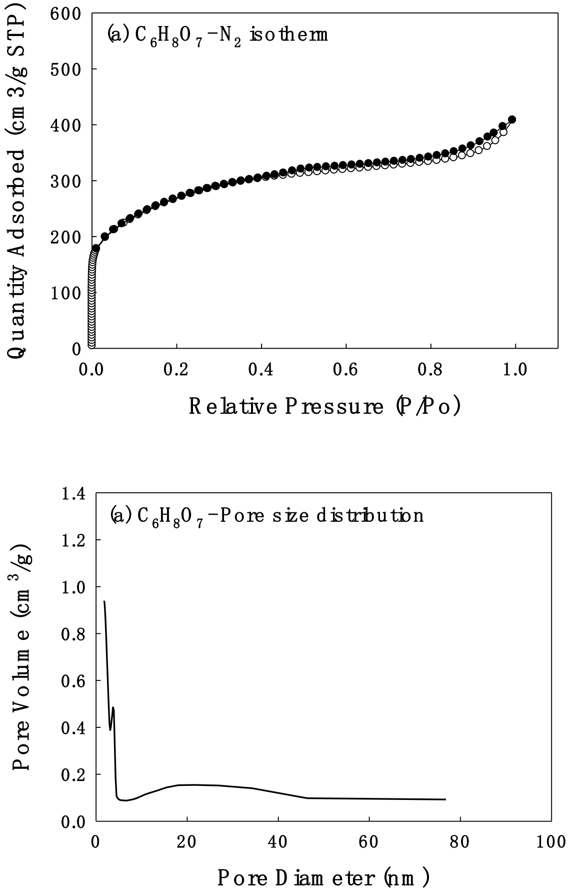

(a) $C_6H_8O_7$

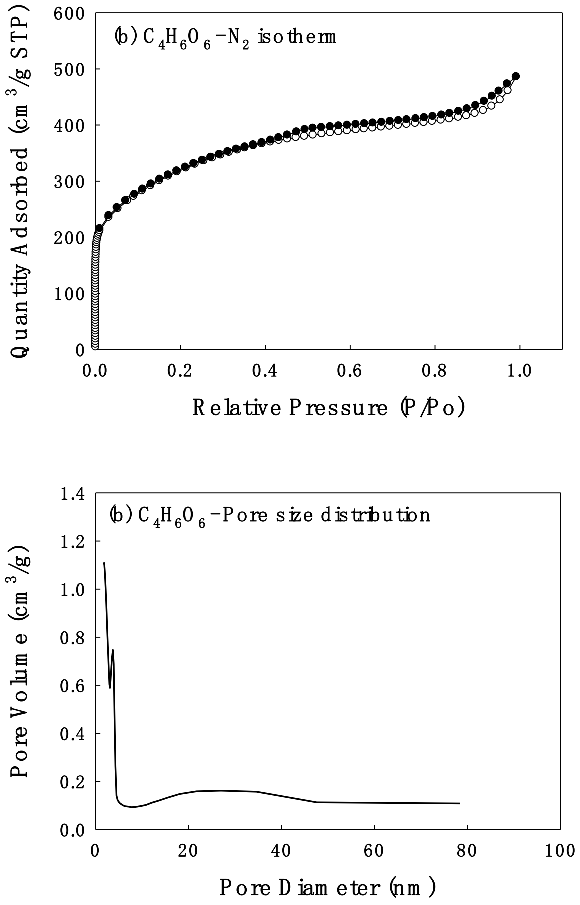

(b) $C_4H_6O_6$

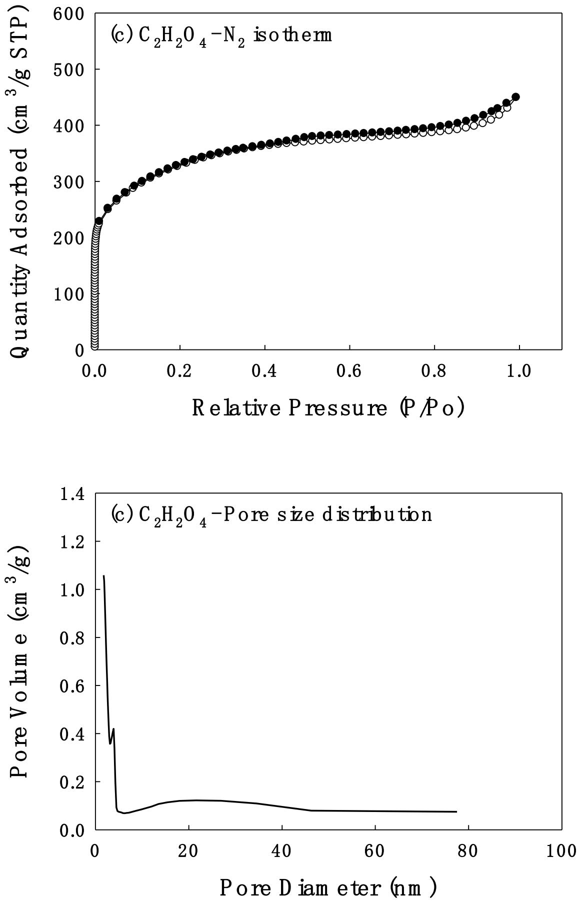

(c) $C_2H_2O_4$

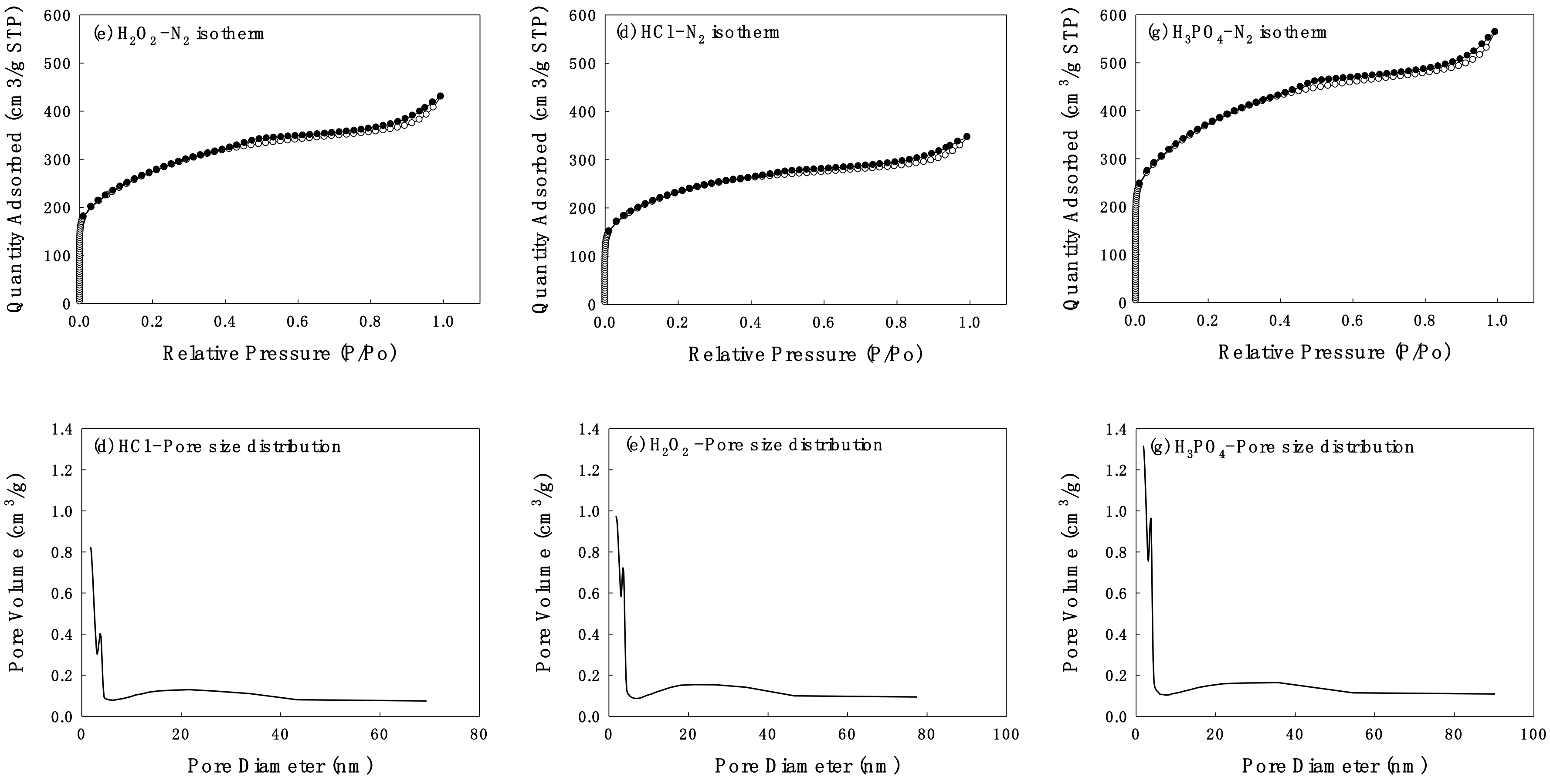

(d) HCl (e)H_2O_2 (f) H_3PO_4

Figure S3. Nitrogen adsorption and desorption isotherms of SAC-A-Cs; (a) $C_6H_8O_7$, (b) $C_4H_6O_6$, (c) $C_2H_2O_4$, (d) HCl, (e)H_2O_2, (f) H_3PO_4.

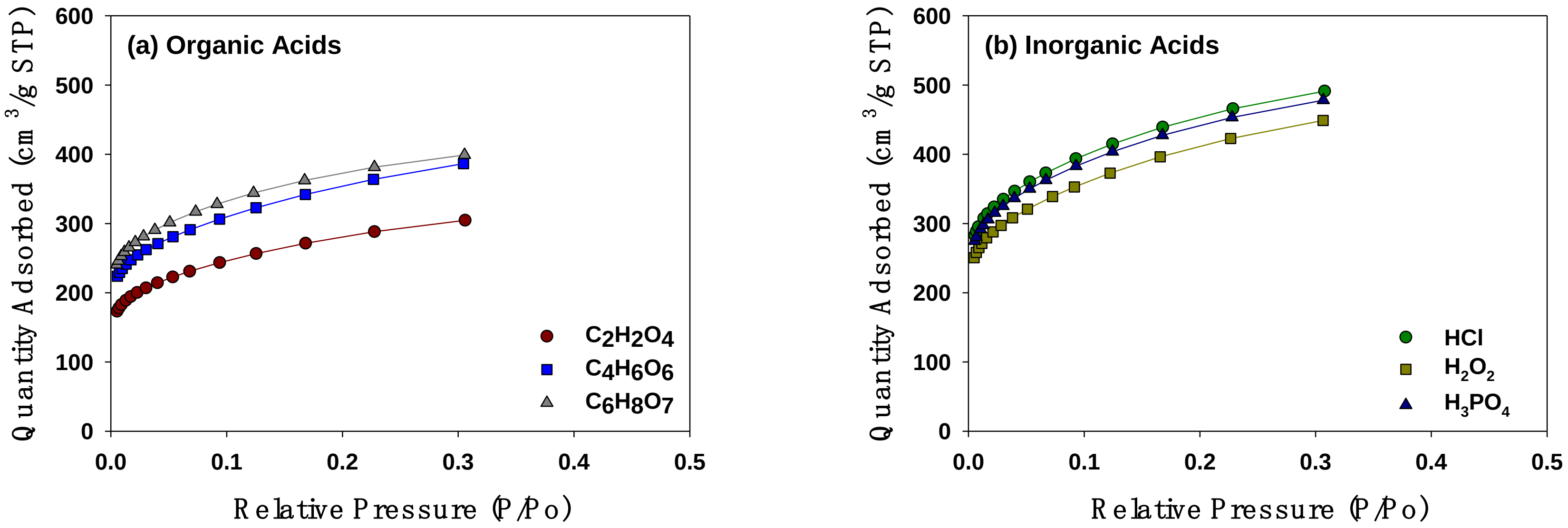

Figure S4. Nitrogen adsorption and desorption isotherms of SAC-H-A-Cs; (a) organic acids; $C_6H_8O_7$, $C_4H_6O_6$, $C_2H_2O_4$, (b) HCl, H_2O_2, H_3PO_4.
